# A Comparative Study of the Effect of TiO_2_ and CuO Nanoparticles as an Additive to Mineral Oil on the Tribological Properties of Steel Friction Pairs

**DOI:** 10.3390/molecules31142450

**Published:** 2026-07-13

**Authors:** Michał Cichomski, Wiktor Stanek, Renata Stanecka-Badura, Magdalena Małecka, Zdzisław Kinart, Joanna Kowalczyk, Monika Anna Madej, Mariusz Dudek

**Affiliations:** 1 Department of Materials Technology and Chemistry, Faculty of Chemistry, University of Lodz, Pomorska 163, 90-236 Lodz, Poland; 2 Doctoral School of Exact and Natural Sciences, University of Lodz, Banacha 12/16, 90-237 Lodz, Poland; 3 FUCHS OIL CORPORATION (PL) Sp. z o.o., Kujawska 102, 44-101 Gliwice, Poland; 4 Department of Physical Chemistry, Faculty of Chemistry, University of Lodz, Pomorska 163/165, 90-236 Lodz, Polandzdzislaw.kinart@chemia.uni.lodz.pl (Z.K.); 5 Faculty of Mechatronics and Mechanical Engineering, Kielce University of Technology, Tysiąclecia Państwa Polskiego 7, 25-614 Kielce, Poland; jkowalczyk@tu.kielce.pl (J.K.);; 6 Institute of Materials Science and Engineering, Lodz University of Technology, Stefanowskiego 1/15, 90-924 Lodz, Poland

**Keywords:** mineral oil, TiO_2_ nanoparticles, CuO nanoparticles, tribology, friction, wear

## Abstract

This study presents the effect of adding crystalline titanium dioxide (TiO_2_) and copper oxide (CuO) nanoparticles to SN mineral oil on the tribological properties of steel samples. X-ray diffraction confirmed the crystal structure of the nanoparticles and atomic force microscopy confirmed their nanometric size (~30 nm). Based on viscosity, surface tension, and finally micro- and macroscale ball-on-disc measurements, the optimal concentration of nanoparticles in oil samples was determined at 0.05 wt.%. The addition of TiO_2_ and CuO nanoparticles to SN oil reduced wear and the coefficient of friction. Moreover, macroscale tests using the XCT tribotester in a cylinder-on-ring configuration showed a higher Brugger value than in SN oil without nanoparticle additives, while a reduction in the coefficient of friction occurred. Analysis of the worn surfaces indicates that the lower coefficient of friction and wear is the result of the formation of protective anti-wear tribofilm containing nanoparticles. The conducted ball-on-disc and Brugger tests confirmed the beneficial effect of TiO_2_ and CuO nanoparticle addition on the tribological properties of the oil and helped explain the mechanism of lubrication of steel surfaces used in friction tests.

## 1. Introduction

Friction, a natural physical phenomenon that has accompanied humans since the dawn of time, and specifically the reduction in energy losses and the extension of the durability of interacting machine components, is one of the most important challenges that humans have faced throughout history. Many strategies exist for achieving low friction coefficients and wear on interacting machine component surfaces [1,2]. These include the production of low-friction coatings based on hydrogenated carbon, molybdenum sulphide, or tungsten sulphide [3,4,5], as well as the production of self-assembled layers [6] of materials such as fluorosilanes [7] and alkylphosphonic acids [8,9]. However, the primary method of reducing friction is external lubrication, a method of reducing friction that involves introducing a lubricant (e.g., mineral or synthetic oils) between interacting surfaces.

To enhance overall lubrication performance, additives such as anti-wear and extreme pressure agents, antifriction compounds, corrosion inhibitors, antioxidants, viscosity index improvers, dispersants, and detergents are commonly employed [10]. Chemical additives like phosphorus, chlorine, and sulphur improve lubrication efficiency by forming a protective chemical layer; however, they also cause adverse environmental effects [11]. Studies indicate that currently used lubricants and various surface modification techniques may fail to ensure sufficient durability [12], functionality [13], and performance of lubricating oils [14]. One potential solution to these issues is the use of lubricants containing nanoparticle additives, commonly referred to as nanolubricants [15,16,17,18,19,20].

Dispersed nanoparticles enhance the tribological properties of lubricants through several mechanisms. These include the polishing effect [20,21], the formation of tribofilms [22], the rolling effect [20,23], and the mending (self-healing) effect [24]. Xu et al. [20] found that paraffin oil containing dispersed diamond nanoparticles reached the frictional interface and transformed sliding friction into rolling friction, effectively acting as micro ball bearings. Using scanning tunnelling microscopy, Liu et al. [24] observed the deposition of copper nanoparticles on worn surfaces, a phenomenon known as the mending effect. Zhou et al. [22] reported that copper nanoparticles interact with the surfaces of friction pairs, forming tribofilms that significantly improve anti-wear performance. Tang and Li [21] noted that the polishing effect produces exceptionally smooth, nearly uniform microplates that reduce surface roughness.

Birleanu et al. [25] indicated that the transfer and adhesiveness of nanoparticles lead to a change in the surface condition and the formation of a thin titanium dioxide (TiO_2_) tribofilm. This leads to a reduction in the coefficient of friction, as well as pressure and temperature, in the contact area.

Another factor influencing the tribological behaviour of nanolubricants is viscosity, which increases with higher nanoparticle concentrations [17,26,27]. Wu et al. [28] investigated the friction-reducing properties of lubricating oils with nano-additives, including CuO, TiO_2_, and diamonds. They found that the base oil containing TiO_2_ nanoparticles exhibited the lowest coefficient of friction, as it had the highest viscosity among the tested lubricants.

Numerous studies in the literature have examined the use of TiO_2_ additives in various oils and their positive impact on tribological properties [29,30,31,32,33,34,35,36,37,38]. It has been proven that the use of low concentrations of TiO_2_ nanoparticles is sufficient to improve tribological characteristics [33]. Nanoparticles at higher concentrations agglomerate more easily in lubricants, which has a detrimental effect, increasing wear and friction [39,40]. Recently, metal oxide nanoparticles such as zinc oxide (ZnO), silicon dioxide (SiO_2_), aluminium oxide (Al_2_O_3_), titanium dioxide (TiO_2_), and copper oxide (CuO) have been favoured as lubricant additives due to their chemical inertness toward oils, allowing the formation of stable colloidal suspensions of nanolubricants [41]. Moreover, the use of ZnO and rGO increased the load-bearing capacity of oils by 30% to 60%, respectively, and reduced the friction coefficient by up to 60% [42]. Consequently, this study focuses on copper oxide and titanium dioxide.

The main objective of this work is to determine the concentration of TiO_2_ and CuO nanoparticles in mineral oil and then compare the effect of the presence of nanoparticles in SN oil on the friction coefficient and wear of steel samples used in friction tests on a micro- and macroscale.

## 2. Results and Discussion

**Figure 1:**
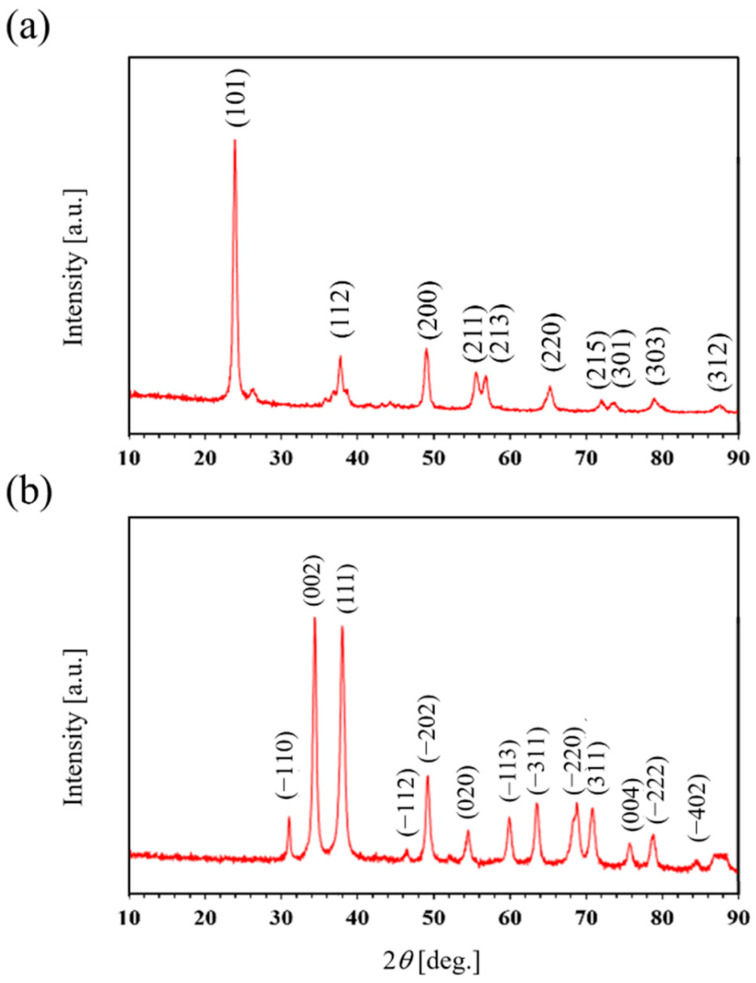
XRD spectra of TiO_2_ (**a**) and CuO powder (**b**).

X-ray diffraction measurement confirmed the crystalline structure of both oxide nanopowders. Figure 1a shows an XRD spectra with peaks characteristic of a tetragonal phase structure of TiO_2_ (JCPDS card no. 00-021-1272), while Figure 1b shows an XRD spectra with peaks characteristic of a monoclinic phase structure of CuO (JCPDS card no. 00-045-0937). Based on Scherrer’s Equation (1), the grain size for the most intense peaks was determined. For TiO_2_ powder, for the peaks from the (101), (112), (200), (211) and (213) planes, the average grain size was 24.2 ± 2.0 nm with a range of variability from 18.8 to 28.9 nm. However, for CuO powder, the values determined for the crystallographic planes (002), (111), (−202), (−113), (−311), and (−220) with a variation range from 24.6 to 48.6 nm gave an average grain size value of 34.2 ± 3.5 nm.

**Figure 2:**
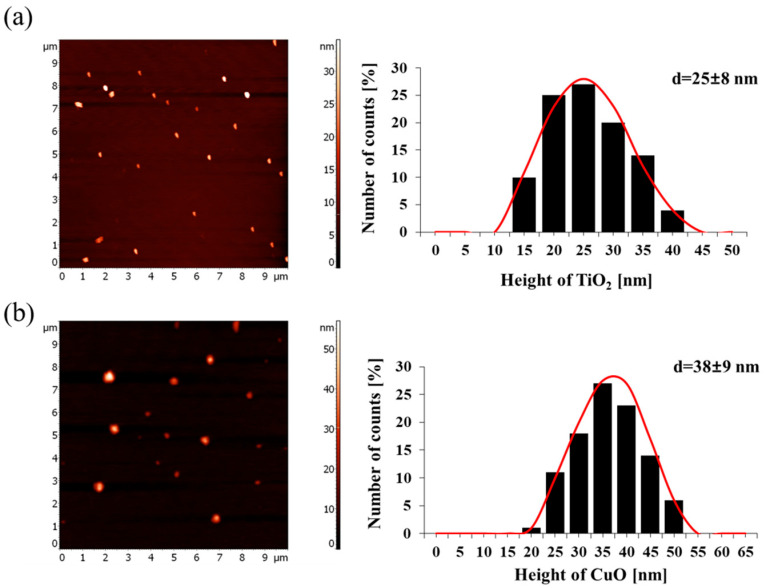
AFM image and particle size distribution of TiO_2_ (**a**) and CuO powder (**b**).

**Figure 3:**
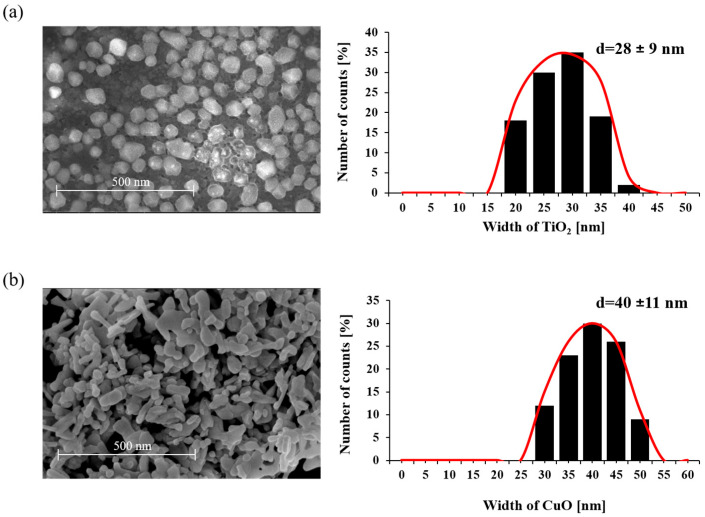
SEM image and particle size distribution of TiO_2_ (**a**) and CuO powder (**b**).

To confirm the nanosize of the oxide powders, the size of the powder particles was measured using an atomic force microscope operating in semi-contact mode and a scanning electron microscope. Figure 2 shows examples of the AFM images of particles deposited on a silicon substrate and histograms of the particle height distributions. For both types of powders, the particle size allows them to be clearly classified as nanomaterials. AFM images show that the objects investigated are polydisperse, without aggregates or agglomerates. The particles are characterised by a large distribution around the mean value: 25 nm and 38 nm for TiO_2_ and CuO nanoparticles, respectively. These values are confirmed by the analysis of SEM images (Figure 3), based on which the average nanoparticle size can be determined at 28 nm for TiO_2_ nanopowder and 40 nm for CuO nanopowder. Considering the XRD measurement results, both powders are monocrystalline nanoparticles. TiO_2_ nanoparticles have an average size of approximately 27.5 nm (in the range of 15–40 nm), while CuO nanoparticles have a size of approximately 39 nm (in the range of 20–50 nm). The smaller sizes of nanoparticles obtained by XRD compared to AFM and SEM are because the former technique only measures crystallites without any sample preparation-related interactions.

**Figure 4:**
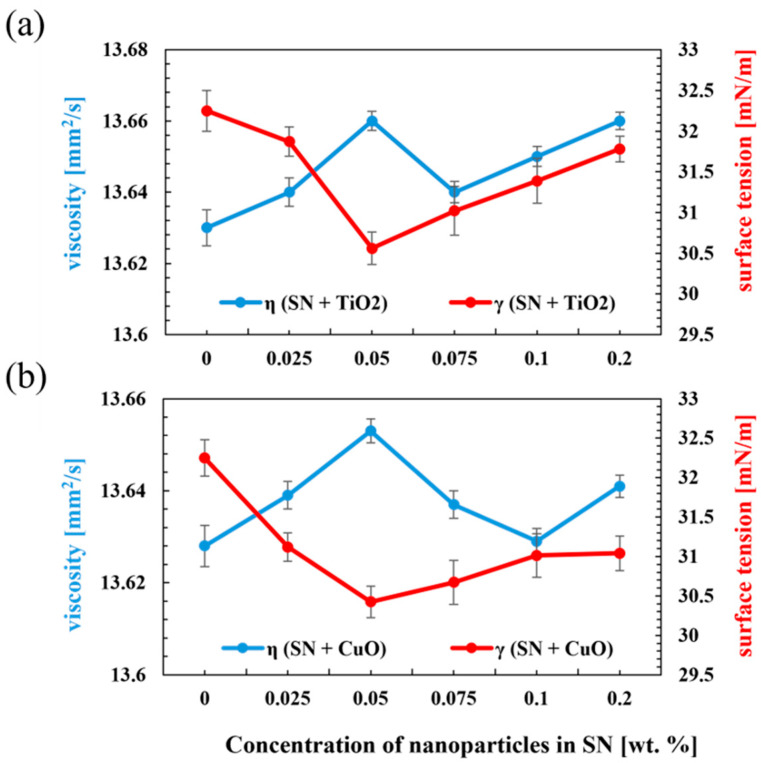
Effect of nanostructure concentration on viscosity and surface tension in SN oil containing TiO_2_ (**a**) and CuO (**b**) nanoparticles.

The optimal concentration of oxide nanoparticles in oil samples was determined based on viscosity and surface tension measurements (Figure 4). Bearing in mind that viscosity depends not only on particle size but also on their chemical composition and spatial arrangement within the particle [43], oil samples were prepared with different concentrations of nanoparticles investigated: 0, 0.025, 0.05, 0.075, 0.1, and 0.2 wt.%. The obtained results (Figure 4) show a clear relationship between viscosity and surface tension: when viscosity reached its maximum, surface tension reached its minimum. This behaviour arises from critical concentration and interfacial tension effects, driven by van der Waals and electrostatic interactions, as noted by Cichomski et al. [44]. A total of 0.05% TiO_2_ and CuO was identified as the optimal concentration, producing maximum viscosity and minimum surface tension, and was deemed appropriate for the tribological tests. It should also be emphasised that, looking at the range of variation (Figure 3), the minimum surface tension values are almost identical in both cases (30.56 and 30.42 mN/m for TiO_2_ and CuO, respectively), while in the case of viscosity, the maximum values differ (13.66 and 13.67 mm^2^/s for TiO_2_ and CuO, respectively).

**Figure 5:**
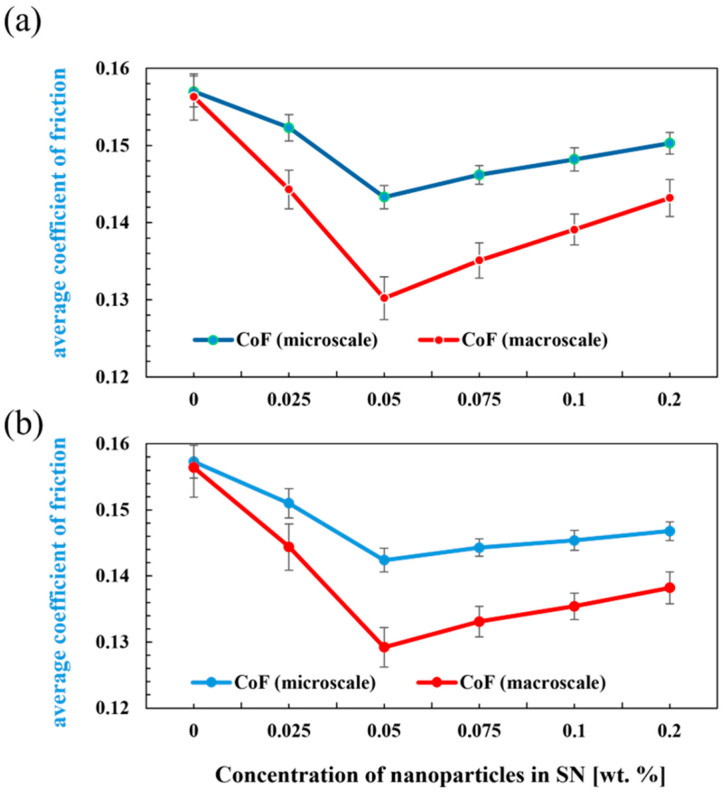
Coefficient of friction after ball-on-disc test in micro- and macroscale as a function of TiO_2_ (**a**) and CuO (**b**) nanoparticle content in SN oil.

To confirm the adopted method for determining the optimal nanoparticle content in SN oil through viscosity and surface tension measurements, ball-on-disc tests were performed for each nanoparticle concentration. Figure 5 presents the results of these tests for micro- (100 mN load) and macroscale (30 N load) conditions. The determined values of the average coefficient of friction confirm that, regardless of the load scale, its minimum occurs at 0.05 wt.% nanoparticle content. Importantly, the change in the coefficient of friction with changing nanoparticle content is similar in its course to changes in surface tension (Figure 4). This emphasises the importance of surface tension measurements for characterising liquid lubricants and determining their suitability under operating conditions.

In the following part of the paper, the lubricant based on SN oil with the optimal content of TiO_2_ nanoparticles (0.05 wt.%) will be designated as SN + TiO_2_, while SN + CuO will designate CuO nanoparticles. Analysing the results of the coefficient of friction for these compositions for both applied loads (Figure 5), its values are significantly lower than for pure SN oil—the presence of nanoparticles reduced the coefficient of friction by approximately 9% at the microscale and by more than 17% at the macroscale compared to the base SN oil. Turning to absolute values (Figure 5), the average value of the coefficient of friction at the microscale for pure SN oil was 0.157. After adding 0.05 wt.% TiO_2_ nanoparticles, it decreased to 0.143, and after adding 0.05 wt.% CuO nanoparticles, it decreased to 0.142. In the case of the macroscale, the coefficient of friction for pure SN oil was 0.156, which decreased to 0.130 after adding TiO_2_, and to 0.129 after adding CuO.

Furthermore, the observed tribological improvements at 0.05 wt.% concentration are closely linked to the kinematic viscosity peaks (13.67 mm^2^/s for TiO_2_ and 13.66 mm^2^/s for CuO) identified during the physicochemical characterisation. The maximum viscosity achieved at this specific concentration likely enhances the load-carrying capacity of the lubricant film.

The kinematic viscosity values for both nanolubricants at the optimal concentration of 0.05 wt.% were found to be essentially identical (13.67 mm^2^/s for TiO_2_ and 13.66 mm^2^/s for CuO). This indicates that the marginal difference in viscosity is not the primary factor responsible for the enhanced performance of CuO. Instead, the superior performance of the SN + CuO mixture in macroscale tests likely results from a more efficient distribution of nanoparticles within the contact zone and the chemically driven formation of a more robust protective tribofilm under higher loads.

The improvement in tribological parameters following the addition of TiO_2_ and CuO nanoparticles likely results from a combination of several mechanisms. The nanoparticles act as microscopic rolling elements, reducing sliding friction, while simultaneously filling the asperities of the steel surfaces (mending effect). This increases the effective contact area and reduces localised contact pressure. The synergy between optimal viscosity, low surface tension, and the presence of nanoparticles allows for the maintenance of a stable lubricant film even under more demanding macroscale conditions, effectively protecting the mating surfaces from direct contact and wear.

**Figure 6:**
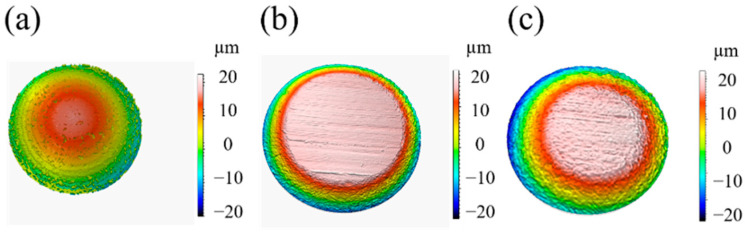
2D maps of balls before (**a**) and after tribological tests with SN (**b**) and SN + CuO (**c**) oil lubrication. Example ball wear maps after ball-on-disc test performed at a load of 100 mN were obtained using a confocal microscope.

**Figure 7:**
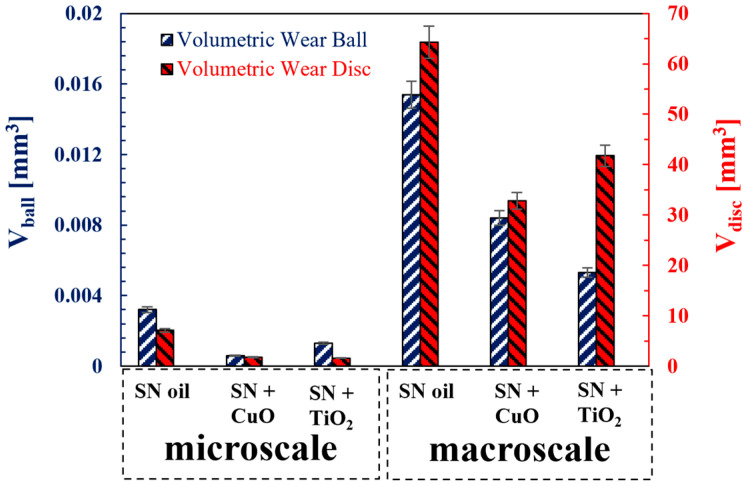
Volumetric wear results of the balls and discs under the load of 100 mN (microscale) and 30 N (macroscale).

Before and after the tribological tests, the surface topography of the balls and discs was examined using a confocal microscope. Figure 6 presents examples of 2D maps for the ball before and after the ball-on-disc test in the microscale. Based on these measurements, the wear volume values of the discs and balls after the ball-on-disc test were determined—the results of these analyses are summarised in Figure 7. As expected, the highest wear occurred in the friction tests for SN oil without nanoparticles. It was at least twice as high as for SN oil with nanoparticles.

First, analysing the friction tests at the microscale (at a load of 100 mN), it can be seen that in the case of the ball, the determined wear volume value for pure SN oil is 0.32 × 10^−2^ mm^3^. For the oil with the addition of TiO_2_ nanoparticles, it decreases to 0.06 × 10^−2^ mm^3^, and for the oil with CuO nanoparticles to 0.13 × 10^−2^ mm^3^. For the disc, these values were 7.116, 1.782, and 1.597 mm^3^, respectively. At the macroscale (load of 30 N), the wear volume was much greater and amounted to 1.54 × 10^−2^ mm^3^ for pure SN oil, 0.84 × 10^−2^ mm^3^ with the addition of TiO_2_ nanoparticles, and 0.53 × 10^−2^ mm^3^ with the addition of Cu nanoparticles. For the disc, these values were 64.293, 32.827, and 41.751 mm^3^. Remaining on the wear of the disc participating in the ball-on-disc test, it should be emphasised that the wear volume for SN oil with TiO_2_ nanoparticles on a microscale is 25% of the wear for pure SN oil, while for CuO nanoparticles it is 22%. On the macroscale, these values are 51% and 64%, respectively. Such large differences between the micro- and macroscale result from different contact mechanisms and dominant physical phenomena. This can already be evidenced by the maximum Hertzian contact pressure for concentrated ball–surface contact. On the microscale, it is 630.8 MPa with a circular contact area diameter of 17 μm, while on the macroscale it is 1954.9 MPa and 171 μm, respectively. The difference in the corresponding parameters for the micro- and macroscales by one order of magnitude is also visible in the specific wear rate determined for the disc (the wear values discussed above were divided by the friction distance and the load value during the ball-on-disc test). As shown in Table 1, the specific wear rate values for the macroscale are one order of magnitude lower than for the microscale.

The ball-on-disc test, applied at two scales, was designed to comprehensively assess the lubricating properties of the tested SN oil with TiO_2_ and CuO nanoparticles. Microscopic tests (circular contact surface diameter equal to 17 μm for concentrated ball–surface contact) allowed for the analysis of phenomena occurring at the level of individual contacts, where surface interactions such as adhesion, van der Waals interactions, and the formation of thin boundary layers dominate. At this scale, surface chemistry and interactions between nanoparticles and the substrate material play a key role. Macroscopic tests (circular contact surface diameter equal to 171 μm for concentrated ball–surface contact) reflect conditions closer to real-world engineering applications, where contact encompasses multiple micro-areas, and the tribological response is the result of averaging processes occurring in a larger volume of material and in the presence of a full lubricant film or mixed lubrication.

The chemical composition and roughness of steel samples were examined before and after the ball-on-disc test. Table 2 summarises the results of chemical composition analyses (contents of Fe, Cr, Si, Mn, Ti and Cu atoms). The absence of Ti and Cu in the chemical composition of the steels from which the ball and disc (respectively 100Cr6 and C45 steel) were made allows us to clearly attribute the presence of TiO_2_ and CuO nanoparticles on the surface of the wear traces formed during the friction test in the presence of lubricant containing TiO_2_ and CuO nanoparticles (Supplementary Materials, Figures S1 and S2). Considering that it has been previously shown that the presence of these nanoparticles in SN oil reduces the coefficient of friction (Figure 6) and wear (Figure 7), it can be clearly stated that these nanoparticles form a protective film [45,46] during the friction test with SN + TiO_2_ and SN + CuO lubricant.

Regardless of the load parameters in ball-on-disc tests, the surface roughness (*S_a_*) in the wear traces increases (Table 3). The largest increase in roughness of the disc occurs for pure SN oil. The presence of nanoparticles in SN oil reduces the increase in roughness in the wear trace. For example, in the case of SN + CuO lubricant, the greatest reduction (tenfold) occurs in the case of the ball under macroscale friction (Table 3). Evidence for this mechanism is provided by the tenfold reduction in ball roughness (Sa) in macroscale tests (from 1.238 µm to 0.126 µm for CuO vs. 0.157 µm for TiO_2_) and the significant presence of Cu (0.66 wt.%) on the wear track, which confirms the formation of a highly effective protective layer unique to the CuO additive.

The presence of CuO nanoparticles resulted in lower wear track roughness and reduced wear during ball-on-disc testing. Also noteworthy are the negative values of the *S_sk_* roughness parameter for friction processes in the presence of SN oil with nanoparticles and before the friction process. This indicates that the topography is dominated by shallow valleys rather than peaks, without the presence of wear-related defects. In summary, the presence of Ti and Cu in the wear trace (Table 2) confirms that TiO_2_ and CuO nanoparticles, used as an anti-wear additive, penetrated the friction surface and became physically adsorbed onto the lubricated interfaces. Such protective layers [45,46] are highly beneficial, as they help reduce the coefficient of friction (Figure 6) and wear (Figure 7). The roughness measurement results also indicate that during the friction test, processes such as the so-called healing effect [47,48] or polishing effect [46,49] also occur.

**Figure 8:**
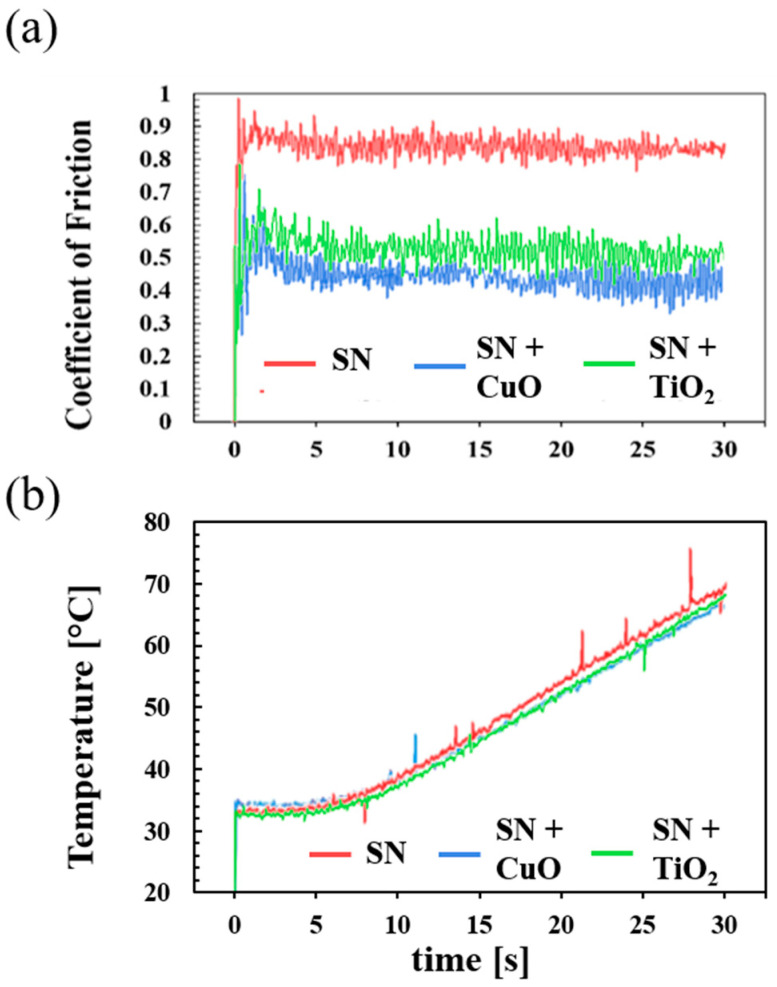
Change in friction coefficient (**a**) and temperature (**b**) during XCT test with pure and contained nanoparticle SN oil.

To evaluate the synergistic effects between SN oil and nano-additives under extreme pressure conditions at the macroscale, the tribological tests were performed using the XCT device (Cross Cylinder Test) in a cylinder on the ring friction pair configuration. Figure 8 presents the tribological characteristics (coefficient of friction and temperature) recorded during the XCT tests for the pure SN oil and the SN oil containing 0.05 wt.% of TiO_2_ and CuO. For pure SN oil, the coefficient of friction initially reached 0.98, then slowly decreased to 0.85 at the end of the test. In the case of SN + TiO_2_ oil, the coefficient of friction, from an initial value of 0.79, stabilised at 0.56. However, for SN + CuO oil, from an initial value of 0.78, it stabilised at 0.51 (Figure 8a).

Comparison of the test results shows that significantly lower coefficient of friction values were achieved when nanoparticles were introduced—representing an improvement of more than 34% and 40% relative to pure SN oil respectively for lubricant containing TiO_2_ and CuO. The presence of nanoparticles in SN oil reduces the rate of the temperature increase during the XCT test (decreases from 1.50 °C/s for pure SN oil to 1.45 °C/s for SN + TiO_2_ and SN + CuO oil). Therefore, during the tests, the maximum temperature for SN oil reached 69 °C, for SN + TiO_2_ it stabilised at 68 °C, while for SN + CuO it stabilised at 67 °C (Figure 8b). These results correlated with the smaller volumetric wear of the cylinder surfaces—62.95, 43.23 and 38.32 mm^3^, respectively, for pure SN oil and with the addition of TiO_2_ and CuO nanoparticles—confirming that the addition of CuO nanoparticles gives the best results (lowest friction coefficient and lowest wear). These results confirm the synergistic effect of CuO nanoparticles within the mineral oil at the sliding interface: the formation of a protective film [45,46], and the so-called healing effect [47,48] or polishing effect [46,49].

To better understand the XCT test results, the Brugger values described by Equation (5) were estimated. For the XCT test with pure SN oil, the Brugger value was 16.95 N/mm^2^. When the test was performed with lubricant containing nanoparticles, the value increased to 24.28 N/mm^2^ for TiO_2_ nanoparticles and to 27.15 N/mm^2^ for CuO nanoparticles. Comparing these results with the results of the volumetric cylinder wear from the previous paragraph (62.95, 43.23 and 38.32 mm^3^, respectively), it can be seen that the greater the increase in the Brugger value, the lower the wear. These results confirmed the beneficial effect of nanomaterial additives (in particular CuO nanoparticles) in improving the performance of the cylinder on the ring tribological system.

**Figure 9:**
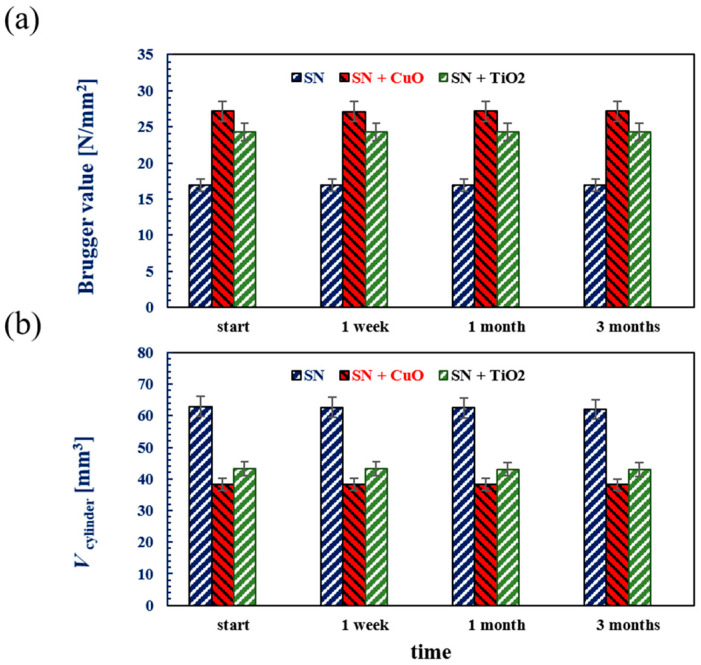
The Brugger value (**a**) and volumetric wear of cylinder (**b**) determined after XCT tests carried out at different times after the preparation of lubricants containing nanoparticles.

The XCT test effectively simulates the friction conditions encountered during metalworking and machining processes, which represent the intended application area for the proposed nanoparticle additives to oil. Therefore, this test was chosen to verify the stability over time of lubricants produced on an SN oil base. Figure 9 shows the Brugger value and volumetric wear of the cylinder determined for tests conducted up to 3 months after the production of the lubricant containing TiO_2_ and CuO nanoparticles. The presented results indicate that the produced lubricants are stable over time.

The lower wear and friction coefficients observed for the CuO-containing oil compared to TiO_2_ are not attributed to the negligible differences in viscosity (Figure 4), which fall within the measurement uncertainty. Rather, these results confirm the superior ability of CuO nanoparticles to interact with the steel surfaces, facilitating a more effective mending effect and the deposition of a copper-rich protective layer, as supported by the lowest Sa values and EDS results.

The presence of copper and titanium elements in the wear scar area, detected by spot EDS analysis, and directly correlated with confocal microscopy results—which showed a reduction in wear volume and microcrack filling—provides strong evidence of a physical mending effect. This indicates local deposition of nanoparticle material in surface depressions rather than the formation of a continuous, chemically bonded tribofilm. This demonstrates only the mending mechanism—the particles entered the microcracks and remained trapped within them. At the same time, the polishing effect was directly confirmed by combined evidence from SEM+EDS photographs and surface profiles analysed by confocal microscopy, which consistently demonstrate the smoother wear scar morphology (*S_a_* and *S_sk_* parameters; Table 3) and the reduction in deep scratches compared to the SN base oil. The change in roughness demonstrates the polishing mechanism. If the roughness decreased, this indicates that polishing occurred. Finally, the rolling effect is indirectly supported by the coefficient of friction curve, where the spherical or quasi-spherical (CuO, TiO_2_) morphology of the nanoparticles promotes the transition from sliding friction to partial rolling friction in the initial stages of contact, before the particles become embedded in the substrate irregularities. The coefficient of friction in Figure 8 demonstrates the rolling effect, where friction dropped dramatically from the very beginning of the test, before the particles had time to become embedded in the metal structure.

## 3. Materials and Methods

### 3.1. Materials

The study employed SN 70 mineral oil (H&R Group, Hamburg, Germany) as the reference lubricant. This oil has a kinematic viscosity of 13.63 mm^2^/s at 40 °C and a density of 0.8569 kg/m^3^ at 15 °C. Crystaline TiO_2_ and CuO nanoparticles were introduced into the base oil. Both oxide nanopowders were supplied by Sigma Aldrich. The materials were used as received without any additional chemical or surface modification. All samples were prepared using analytical-grade reagents.

### 3.2. Nanoparticle Characterisation

The morphology and size distribution of nanoparticles were determined by scanning electron microscope (SEM), atomic force microscope (AFM) and X-ray diffraction (XRD). The scanning electron microscopic analysis was performed on a Schottky Field Emission Scanning Electron Microscope (FESEM), FEI apparatus, model Nova NanoSEM 450 (Hillsboro, OR, USA). Images were acquired in the immersion mode using a through-lens detector (TLD) at a magnification of 100,000×. AFM analysis was performed using a Solver P47, NT-MDT instrument (NT-MDT, Moscow, Russia). Samples were prepared by dispersing nanopowder in a volatile solvent, followed by drop-casting onto a silicon substrate and drying under ambient conditions. Measurements were conducted in semi-contact mode. The tests were carried out for an image size of 10 × 10 mµ. Particle size (height) analysis was performed using the manufacturer’s software. The average sizes of NPs were determined by measuring at least one hundred objects and creating a histogram. Next, the size distribution histogram was created, and for each data set the mean size and standard deviation were calculated. XRD measurements were performed using an Aeris 1.2.0 (Almelo, Nederland) PANalytical diffractometer equipped with a PIXcel detector and Cu radiation (CuKα, *λ* = 0.154178 nm). Measurements were conducted over a 2θ range of 10–90° at a scan rate of 0.9°/s. The grain size (*D*_*hkl*_) of the nanoparticles was calculated using Scherrer’s Equation:

(1) $$D_{hkl}=\frac{K\;\lambda }{\beta \cos\theta_{hkl}\;}\;$$

where *K* is Scherrer’s constant (taken as 0.9), *β* is the full width at half maximum (FWHM) of the diffraction peak, *θ_hkl_* is the diffraction angle.

### 3.3. Preparation of Nanolubricants

Nanolubricants were prepared by dispersing TiO_2_ and CuO nanoparticles in SN70 base SN oils at concentrations of 0, 0.25, 0.05, 0.075, 0.1, and 0.2 wt.%. The required amount of nanomaterial was accurately weighed on a precision electronic balance and added to the base oil. Ultrasonic agitation (sonication) was then applied for 30 min to disperse the nanoparticles. The prepared samples (nanofluids were placed in glass bottles and sealed tightly) were stored for 7 days to ensure that stable oxide nanomaterial suspensions formed in the mineral-oil-based nanofluids before further testing. After confirming that no nanoparticle precipitation occurred, physicochemical analyses (viscosity and surface tension) of the prepared oil samples began. The stability of the nanofluids was assessed by observing the deposition (sedimentation) of CuO nanoparticles and TiO_2_ in SN oil for 3 months according to the procedure proposed by Lee and Mudawar [50].

### 3.4. Physicochemical Measurements

Kinematic viscosity of nanofluids was measured at *T* = 40 °C using capillary viscometers with standard procedures for lubricating oils. Temperature was controlled within ±0.1 °C. Surface tension measurements were performed with a KSV Sigma 701 (Hamburg, Germany) tensiometer. The measurement procedure relied on determining the force acting between the probe (a platinum Wilhelmy plate) and the interface between the test liquid and the gas phase (air) at ambient temperature, following the method described in [43]. The reported surface tension values represent the mean of at least three measurements.

### 3.5. Tribological Testing

Tribological tests were carried out using a ball–disc configuration on two testing systems: the NTR^3^ nano tribometer (Anton Paar, Graz, Switzerland) and the T-11 tribometer (Institute for Sustainable Technologies, Radom, Poland). The disc specimens, made of C45 steel with a diameter of 25.4 mm, had a hardness of 30–35 HRC. The balls, fabricated from 100Cr6 bearing steel, measured 2.00 mm (microscale test) and 6.35 mm (macroscale test) in diameter and had a hardness of 60–62 HRC. All tests were performed under ambient conditions (temperature 24 ± 4 °C; relative humidity 37 ± 15%). The test parameters included a normal load of 100 mN (microscale) and 30 N (macroscale), a constant sliding speed of 0.1 m/s, and a total sliding distance of 1000 m.

To further evaluate lubricating performance, Brugger tests were conducted according to DIN 51347-1 and 51347-2, using the XCT (Cross Cylinder Test) tribotester (Mannheim, Germany) in a cylinder on ring configuration. The test was performed in concentrated lubricated sliding contact between a rotating ring (Ø = 25 mm, hardness 60 HRC, surface roughness *R_a_* < 0.8 μm) and a stationary cylinder (Ø = 18 mm, hardness 65 HRC, *R_a_* < 0.2 μm), both made of 100Cr6 bearing steel (AISI 52100) (Brema, Germany). The test consisted of measuring the friction level when the ring was pressed against the cylinder under a 400 N load for 30 s at 960 rpm. The cylinder surface was covered with 8 ± 0.5 mL of the tested lubricant before each run. During testing, the coefficient of friction and cylinder temperature were continuously monitored. After each test, the width of the wear scar on the cylinder was measured. Before subsequent tests, the ring was polished to restore surface smoothness, and the cylinder was rotated to expose an unworn area for contact. The XCT tests were conducted up to 3 months after the production of the lubricant containing TiO_2_ and CuO nanoparticles to confirm their stability over time.

### 3.6. Wear and Surface Analysis

After tribological tests the wear track surface area was examined using a Leica DCM8 confocal microscope (Wetzlar, Germany), (with ×50 objective lens magnification). The analysed area was 0.95 mm × 0.71 mm for the balls and 0.95 mm × 0.26 mm for the discs. The measurement areas were obtained by stitching several individual images—4 × 4 for the balls and 4 × 1 for the discs, respectively. The analysis of 3D surface maps was performed using Leica Map 360 software 2025.0.0 [51]:

(2) $$V_{spheres}=\frac{1}{3}h^{2}\left(3R-h\right)$$

(3) $$h=R-\sqrt{R^{2}-r^{2}}$$

where *r* is the radius of the wear path, *h* is the height of the worn surface, and *R* is the radius of the ball. However, the volumetric wear of the discs was determined by multiplying the area of the wear mark and the length of the wear track.

In the case of Brugger tests, the volumetric wear of the cylinder was determined based on the equation:

(4) $$V_{cylinder}=\frac{4}{3}\pi abh$$

where *a*, *b* are dimensions of axis *a* and axis *b* concerning the wear marks on the cylinder surface, and *h* is wear mark depth. The Brugger value was also determined as the ratio of the applied normal load (*F*) to the worn surface area, in accordance with the formula:

(5) $$B=\frac{4\times F}{a\times b\times \pi }$$

Based on the surface maps obtained using a confocal microscope, the following parameters were also determined for the surfaces involved in the friction tests: *S_a_*—arithmetic mean deviation of the height of surface irregularities from the reference plane; and *S_sk_*—skewness coefficient of the surface topography heights (ordinates) distribution. These values were determined in accordance with the ISO 25178 standard (Geneva, Switzerland) [52].

### 3.7. SEM–EDS Analysis

The chemical composition of the contact surfaces was analysed before and after testing using a Phenom XL scanning electron microscope equipped with an EDS detector to characterise lubrication mechanisms in detail. The measurements were carried out at an accelerating voltage of 15 kV with ×5000 magnification.

## 4. Conclusions

The TiO_2_ and CuO nanoparticles with a structure and size (~30 nm) confirmed by XRD and AFM studies (respectively) were added to SN mineral oil. Based on viscosity and surface tension measurements, the optimal concentration of nanoparticles in SN oil samples was determined at 0.05 wt.%. Ball-on-disc tests at the microscale (100 mN load) and macroscale (30 N load) confirmed that the minimum value of the coefficient of friction occurs at a nanoparticle content of 0.05 wt.%. The correlation between the coefficient of friction and surface tension with the change in nanoparticle content in SN oil indicates the importance of surface tension measurements for characterising liquid lubricants and determining their suitability under operating conditions.

The comparison of the friction results for the ball-on-disc test shows that the presence of nanoparticles reduced the coefficient of friction by approximately 9% at the microscale and by more than 17% at the macroscale compared to the base SN oil. Chemical composition analysis of the tribological pairs indicated that the use of oils containing nanoparticles led to the formation of a protective anti-wear tribofilm on the sliding surface.

Macroscale tests using the XCT tribotester in a cylinder-on-ring configuration showed that lower coefficient of friction values were achieved when nanoparticles were introduced—representing an improvement of more than 34% and 40% relative to pure SN oil respectively for lubricants containing TiO_2_ and CuO nanoparticles. The XCT test results, which remained unchanged over time (up to 3 months after lubricant preparation), confirmed the positive influence of the additives on the performance of the tribological system under operating conditions typical of machining applications.

In summary, adding nanoparticles to SN mineral oil (0.05 wt.%) reduces the coefficient of friction and wear during micro and macro friction tests. The study demonstrates that CuO nanoparticles provide lower friction and wear values than TiO_2_ nanoparticles at the same optimal concentration (0.05 wt.%). Since the viscosity of both nanolubricants is practically identical, the superior performance of CuO is attributed to its enhanced synergistic action, involving the rolling effect, more effective filling of surface asperities (mending), and the formation of a stable, protective Cu-containing tribofilm.

## Figures and Tables

**Table 1:** Specific wear rate values determined for the disc participating in the ball-on-disc test.

Lubricant	Specific Wear Rate [mm^3^ m^−1^ N^−1^]	
	Microscale	Macroscale
SN oil	0.72 × 10^−1^	0.21 × 10^−2^
SN + TiO_2_ oil	0.18 × 10^−1^	0.11 × 10^−2^
SN + CuO oil	0.16 × 10^−1^	0.14 × 10^−2^

**Table 2:** Chemical composition of steel surfaces before and after the ball-on-disc test with two loads.

Lubricant	Sample	Element [wt.%]					
		Fe	Cr	Si	Mn	Ti	Cu
Before test	Ball	97.78	1.49	0.48	0.25	-	-
	Disc	97.63	1.16	0.62	0.59	-	-
**Microscale test (100 mN)**							
SN oil	Disc	97.33	0.99	0.89	0.79		
SN + TiO_2_ oil	Disc	96.69	1.26	0.98	0.65	0.42	
SN + CuO oil	Disc	96.97	1.12	0.78	0.69		0.44
**Macroscale test (30 N)**							
SN oil	Disc	96.62	2.47	0.58	0.33		
SN + TiO_2_ oil	Disc	97.23	1.58	0.80	0.28	0.11	
SN + CuO oil	Disc	97.53	0.92	0.59	0.30		0.66

**Table 3:** Surface roughness parameters of steel discs and balls before and after tribological tests at different loads.

Lubricant	Sample	Roughness Parameters	
		*S_a_* [µm]	*S_sk_* [-]
Before test	Ball	0.026	−0.494
	Disc	0.090	−1.198
**Microscale test (100 mN)**			
SN oil	Ball	3.083	0.015
	Disc	1.049	−0.423
SN + TiO_2_ oil	Ball	2.789	0.014
	Disc	0.861	−0.297
SN + CuO oil	Ball	2.419	−0.137
	Disc	0.895	−0.425
**Macroscale test (30 N)**			
SN oil	Ball	1.238	0.445
	Disc	1.114	0.659
SN + TiO_2_ oil	Ball	0.157	−0.503
	Disc	1.004	−0.041
SN + CuO oil	Ball	0.126	−0.013
	Disc	0.838	−1.828

## Data Availability

The original contributions presented in this study are included in the article. Further inquiries can be directed to the corresponding author.

## Supplementary Materials

The following supporting information can be downloaded at: https://www.mdpi.com/article/10.3390/molecules31142450/s1, Figure S1: The SEM images of the surface and characteristic spectra (EDS) in selected micro-areas along the wear traces of the discs as a result of contact of friction pairs during lubrication with (a) SN oil under a load of 100 mN, (b) SN oil under a load of 30 N, (c) SN + TiO_2_ oil under a load of 100 mN, and (d) SN + TiO_2_ oil under a load of 30 N. Figure S2: The SEM images of the surface and characteristic spectra (EDS) in selected micro-areas along the wear traces of the discs as a result of contact of friction pairs during lubrication with (a) SN oil under a load of 100 mN, (b) SN oil under a load of 30 N, (c) SN + CuO oil under a load of 100 mN, and (d) SN + CuO oil under a load of 30 N.
