# Supplementary material for: A Comparative Study of the Effect of TiO2 and CuO Nanoparticles as an Additive to Mineral Oil on the Tribological Properties of Steel Friction Pairs
Source: Molecules. 2026 Jul 13;31(14):2450. doi: 10.3390/molecules31142450 (PMC13415176; doi:10.3390/molecules31142450)
Supplement: Supplementary file 1 [file molecules-31-02450-s001.zip › molecules-4303335-supplementary.pdf]

Supplementary material

A Comparative Study of the Effect of TiO_2 and CuO Nanoparticles as an Additive to Mineral Oil on the Tribological Propeties of Steel Friction Pairs

Michał Cichomski [1,*], Wiktor Stanek [1,2,3], Renata Stanecka–Badura [1], Magdalena Małecka [4], Zdzisław Kinart [4], Joanna Kowalczyk [5], Monika Madej [5], Mariusz Dudek [6]

[1] Department of Materials Technology and Chemistry, Faculty of Chemistry, University of Lodz, Pomorska 163, 90-236 Lodz, Poland; renata.stanecka.badura@chemia.uni.lodz.pl (R.SB.); wiktor.stanek@edu.uni.lodz.pl (W.S.)
[2] University of Lodz, Doctoral School of Exact and Natural Sciences, Banacha 12/16, 90-237 Lodz, Poland
[3] FUCHS OIL CORPORATION (PL) Sp. z o.o., Kujawska 102, 44-101 Gliwice, Poland
[4] Department of Physical Chemistry, Faculty of Chemistry, University of Lodz, Pomorska 163/165, 90-236 Lodz, Poland; magdalena.malecka@chemia.uni.lodz.pl (Ma.M.); zdzislaw.kinart@chemia.uni.lodz.pl (Z.K)
[5] Faculty of Mechatronics and Mechanical Engineering, Kielce University of Technology, Tysiąclecia Państwa Polskiego 7, 25-614 Kielce, Poland; jkowalczyk@tu.kielce.pl (J.K.); mmadej@tu.kielce.pl (M.M.)
[6] Institute of Materials Science and Engineering, Lodz University of Technology, Stefanowskiego 1/15, 90-924 Lodz, Poland; mariusz.dudek@p.lodz.pl (M.D.);

* Correspondence: michal.cichomski@chemia.uni.lodz.pl (M.C.); Tel.: +48-42-635-58-36

a)

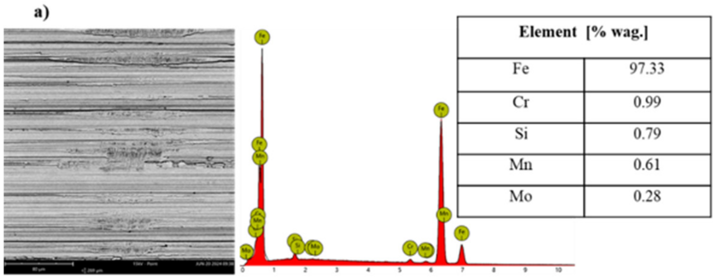

Element	[% wag.]
Fe	97.33
Cr	0.99
Si	0.79
Mn	0.61
Mo	0.28

b)

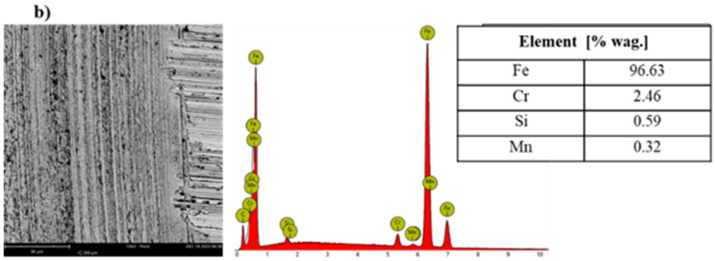

Element	[% wag.]
Fe	96.63
Cr	2.46
Si	0.59
Mn	0.32

c)

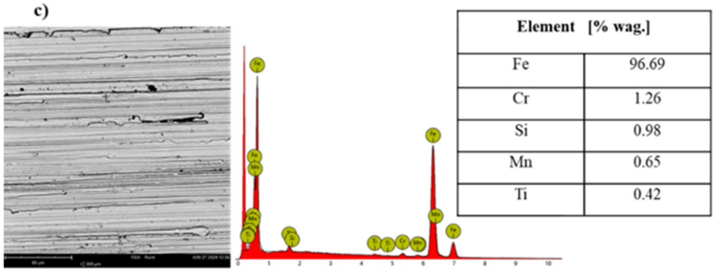

Element	[% wag.]
Fe	96.69
Cr	1.26
Si	0.98
Mn	0.65
Ti	0.42

d)

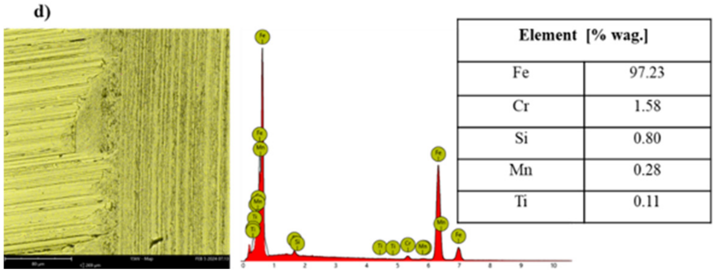

Element	[% wag.]
Fe	97.23
Cr	1.58
Si	0.80
Mn	0.28
Ti	0.11

Figure S1. The SEM images of the surface and characteristic spectra (EDS) in selected micro-areas along the wear traces of the discs as a result of contact of friction pairs during lubrication with a) SN oil under a load of 100 mN, b) SN oil under a load of 30 N, c) SN + TiO_2 oil under a load of 100 mN, and d) SN + TiO_2 oil under a load of 30 N.

a)

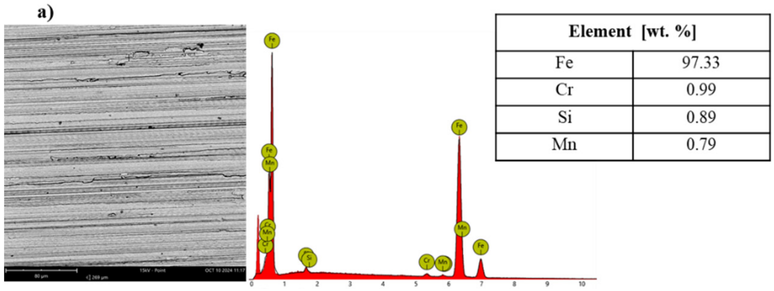

Element	[wt. %]
Fe	97.33
Cr	0.99
Si	0.89
Mn	0.79

b)

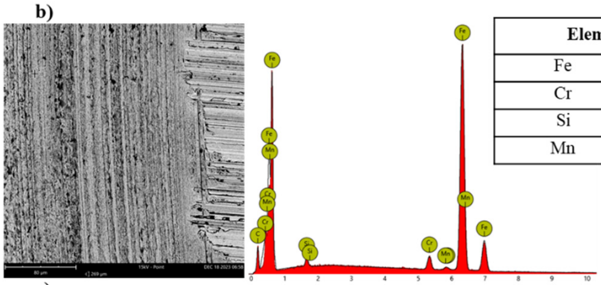

Element	[wt. %]
Fe	96.62
Cr	2.47
Si	0.58
Mn	0.33

c)

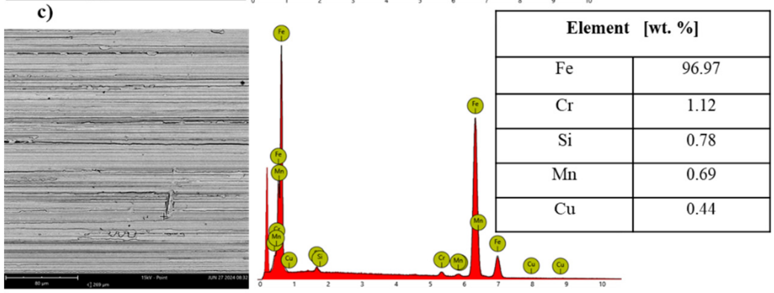

Element	[wt. %]
Fe	96.97
Cr	1.12
Si	0.78
Mn	0.69
Cu	0.44

d)

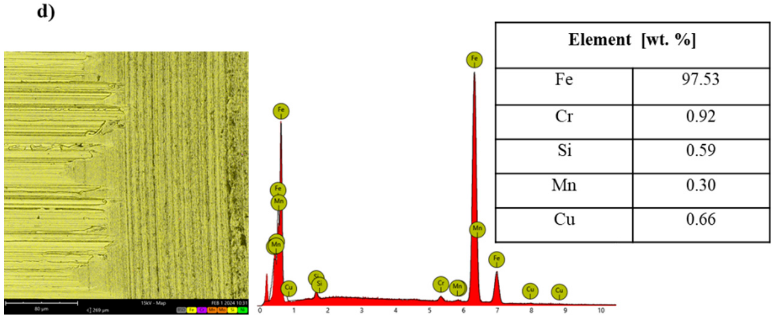

Element	[wt. %]
Fe	97.53
Cr	0.92
Si	0.59
Mn	0.30
Cu	0.66

Figure S2 The SEM images of the surface and characteristic spectra (EDS) in selected micro-areas along the wear traces of the discs as a result of contact of friction pairs during lubrication with a) SN oil under a load of 100 mN, b) SN oil under a load of 30 N, c) SN + CuO oil under a load of 100 mN, and d) SN + CuO oil under a load of 30 N.
